# Spatial imaging features derived from SUVmax location in resectable NSCLC are associated with tumor aggressiveness

**DOI:** 10.1007/s00259-025-07528-0

**Published:** 2025-08-21

**Authors:** Zewen Jiang, Clemens Spielvogel, David Haberl, Josef Yu, Maximilian Krisch, Szabolcs Szakall, Peter Molnar, Janos Fillinger, Lilla Horvath, Ferenc Renyi-Vamos, Clemens Aigner, Balazs Dome, Christian Lang, Zsolt Megyesfalvi, Lukas Kenner, Marcus Hacker

**Affiliations:** 1 Department of Biomedical Imaging and Image-Guided Therapy, Division of Nuclear Medicine, Medical University of Vienna, Währinger Gürtel 18-20, Vienna, 1090 Austria; 2 Christian Doppler Laboratory of Applied Metabolomics, Medical University of Vienna, Vienna, Austria; 3 Pozitron PET/CT Center, Budapest, Hungary; 4 National Koranyi Institute of Pulmonology, Budapest, Hungary; 5 Department of Thoracic Surgery, National Institute of Oncology-Semmelweis University, Budapest, Hungary; 6 National Institute of Oncology and National Tumor Biology Laboratory, Budapest, Hungary; 7 Department of Thoracic Surgery, Comprehensive Cancer Center, Medical University of Vienna, Vienna, Austria; 8 Department of Translational Medicine, Lund University, Lund, Sweden; 9 Division of Pulmonology, Department of Medicine II, Medical University of Vienna, Vienna, Austria; 10 Clinical Institute of Pathology, Medical University of Vienna, Vienna, Austria; 11 Unit of Laboratory Animal Pathology, University of Veterinary Medicine Vienna, Vienna, Austria; 12 Department of Molecular Biology, Umeå University, Umeå, Sweden; 13 Comprehensive Cancer Center, Medical University Vienna, Vienna, Austria

**Keywords:** NSCLC, Tumor invasiveness, [^18^F]FDG PET/CT, Radiomics

## Abstract

**Purpose:**

Accurate non-invasive prediction of histopathologic invasiveness and recurrence risk remains a clinical challenge in resectable non-small cell lung cancer (NSCLC). We developed and validated the Edge Proximity Score (EPS), a novel [^18^F]FDG PET/CT-based spatial imaging feature that quantifies the displacement of SUVmax relative to the tumor centroid and perimeter, to assess tumor aggressiveness and predict progression-free survival (PFS).

**Methods:**

This retrospective study included 244 NSCLC patients with preoperative [^18^F]FDG PET/CT. EPS was computed from normalized SUVmax-to-centroid and SUVmax-to-perimeter distances. A total of 115 PET radiomics features were extracted and standardized. Eight machine learning models (80:20 split) were trained to predict lymphovascular invasion (LVI), visceral pleural invasion (VPI), and spread through air spaces (STAS), with feature importance assessed using SHAP. Prognostic analysis was conducted using multivariable Cox regression. A survival prediction model incorporating EPS was externally validated in the TCIA cohort. RNA sequencing data from 76 TCIA patients were used for transcriptomic and immune profiling.

**Results:**

EPS was significantly elevated in tumors with LVI, VPI, and STAS (*P* < *0.001*), consistently ranked among the top SHAP features, and was an independent predictor of PFS (HR = 2.667, *P* = 0.015). The EPS-based nomogram achieved AUCs of 0.67, 0.70, and 0.68 for predicting 1-, 3-, and 5-year PFS in the TCIA validation cohort. High EPS was associated with proliferative and metabolic gene signatures, whereas low EPS was linked to immune activation and neutrophil infiltration.

**Conclusion:**

EPS is a biologically relevant, non-invasive imaging biomarker that may improve risk stratification in NSCLC.

**Supplementary Information:**

The online version contains supplementary material available at 10.1007/s00259-025-07528-0.

## Introduction

Non-small cell lung cancer (NSCLC) is the leading primary lung malignancy and remains one of the most common causes of cancer-related death globally [1, 2]. Despite the development of new therapies, including targeted treatments and immunotherapy, the prognosis for patients diagnosed with NSCLC remains dismal. The five-year survival rates for those with advanced NSCLC range from about 6–15% [3]. Although early-stage NSCLC is potentially curable with surgical resection, recurrence occurs in approximately 30–50% of patients, most commonly within the first three years after surgery [4]. Prognostic variability in NSCLC is influenced by several critical factors [5, 6]. The tumor-node-metastasis (TNM) staging system serves as the traditional basis for risk stratification; it is increasingly recognized that this system alone does not capture the complete biological behaviors of the disease nor reliably predict recurrent disease, particularly in early-stage patients [7].

The invasive behavior of NSCLC is reflected in several histopathologic patterns, notably visceral pleural invasion (VPI) and lymphovascular invasion (LVI), and it spreads through air spaces (STAS). Although these features involve anatomically distinct pathways, they share common biological characteristics associated with aggressive tumor progression [8, 9]. VPI occurs when tumor cells breach the elastic layer of the visceral pleura, a process often facilitated by degradation of the extracellular matrix and cell changes, cell adhesion [10]. LVI refers to tumor cells within lymphatic or blood vessels and is frequently linked to epithelial–mesenchymal transition (EMT), which enhances cellular motility [11]. STAS, defined as the presence of tumor cell clusters within alveolar spaces beyond the main tumor margin, also indicates detachment and spread, and is associated with reduced intercellular adhesion and increased invasiveness [12, 13]. These invasion patterns frequently co-occur and have been independently associated with nodal metastasis, early recurrence, and worse survival outcomes [14], which shows their significance in prognostication and postoperative risk stratification [15, 16].

Positron emission tomography/computed tomography (PET/CT) with 2-deoxy-2-[^18^F] fluoro-D-glucose ([^18^F] FDG) is widely recommended for the initial staging of NSCLC and plays a central role in treatment planning and prognostic evaluation [17]. Radiomics, the high-throughput extraction of quantitative features from imaging data, emerges as a powerful marker in assessing tumor aggressiveness in NSCLC [18]. Studies have demonstrated that texture, intensity, and shape-based features extracted from PET/CT scans can non-invasively predict tumor characteristics, including VPI [19], LVI [20], and STAS [21]. More recently, attention has turned to spatial imaging features that describe the geometric distribution of metabolic activity within the tumor. In NSCLC, the spatial distance between maximum standardized uptake values (SUVmax) in different lesions has been shown to reflect tumor dissemination and metabolic heterogeneity, offering prognostic value [22]. Within individual tumors, two intra-lesional spatial features, normalized distance from SUVmax to the tumor centroid (nDmaxC) and the normalized distance to the tumor perimeter (nDmaxP), have demonstrated significant prognostic relevance [23]. These features are robust across imaging parameters, complementary to conventional PET metrics, and have independent prognostic value, effectively stratifying outcomes across different treatment settings [24]. However, relying on a single spatial metric can be insufficient. For example, a large distance between SUVmax and the centroid does not always indicate proximity to the tumor edge. To address this limitation, we developed a novel composite parameter, the Edge Proximity Score (EPS), which integrates both nDmaxC and nDmaxP to capture peripheral metabolic activity better.

In this study, we aimed to evaluate EPS as a spatial imaging biomarker in NSCLC by assessing its association with tumor invasiveness, recurrence risk, and key radiomic features. We also explored its biological and clinical relevance through transcriptomic and immune profiling to gain mechanistic insights into the imaging phenotype and its potential application in risk stratification.

## Materials and methods

This retrospective multicenter study included patients diagnosed with NSCLC who underwent surgical tumor resection at two institutions: the Vienna General Hospital (Vienna, Austria) and the National Korányi Institute of Pulmonology (Budapest, Hungary), between January 1, 2010, and December 1, 2020. The study protocol was approved by the Institutional Review Board of the Medical University of Vienna (approval number: 1649/2016) and the Research Committee of the Hungarian Medical Research Council (approval number: 52614–4/2013/EKU), with waivers of informed consent granted due to the retrospective nature of the study.

### Study sample

**Fig. 1:**
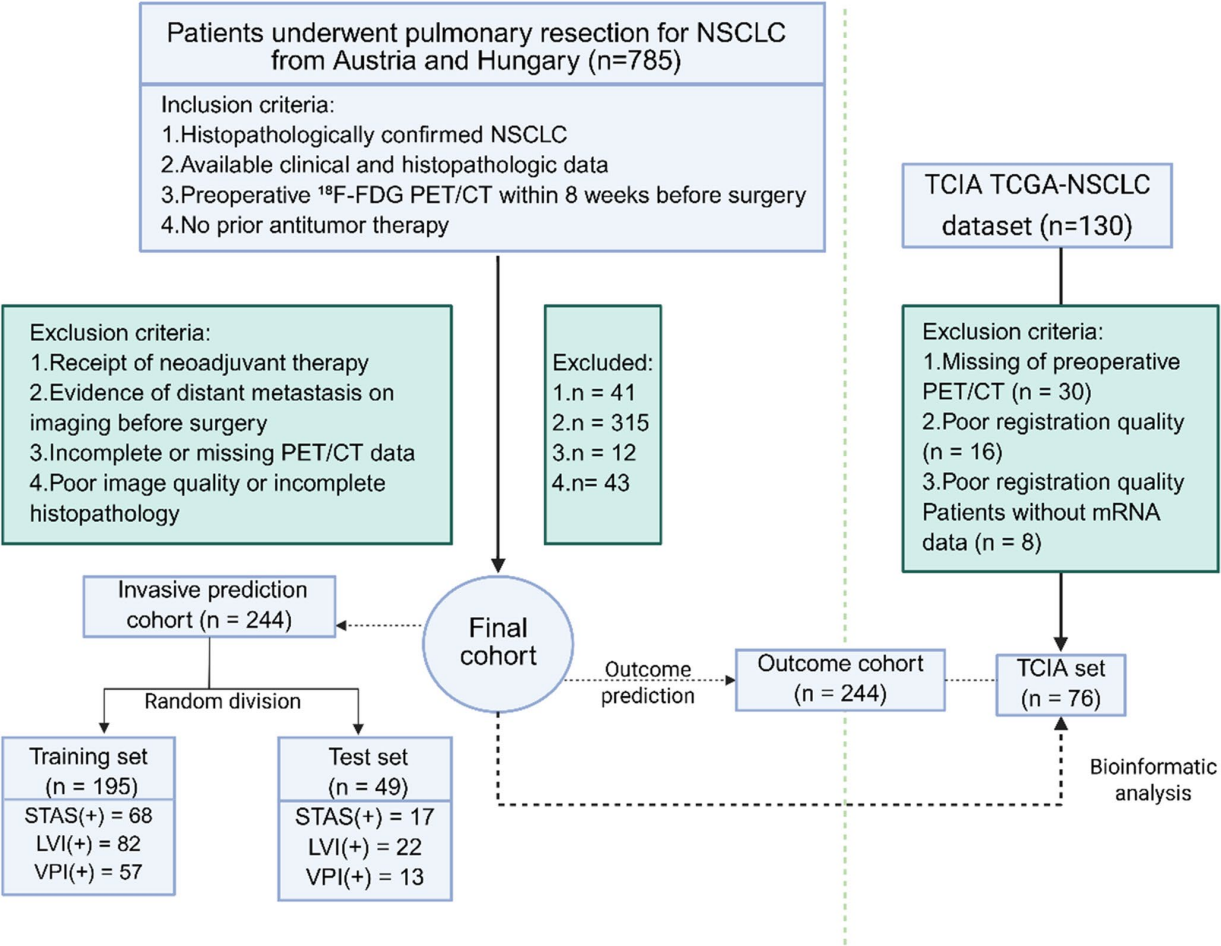
Study design and patient selection workflow. Bioinformatic analysis refers to transcriptomic profiling, pathway enrichment analysis, and immune cell infiltration evaluation. NSCLC, non–small cell lung cancer; VPI, visceral pleural invasion; STAS, spread through air spaces; LVI, lymphovascular invasion; TCIA, The Cancer Imaging Archive; TCGA, The Cancer Genome Atlas

A total of 915 NSCLC patients from three centers, Vienna (*n* = 684), Budapest (*n* = 101), and The Cancer Imaging Archive (TCIA, *n* = 130) [25], were considered for inclusion (Fig. 1). Considering the differences in sample size, tumor stage, and histologic subtype between the Vienna and Budapest cohorts, we merged both institutional datasets into a single cohort to avoid confounding and ensure more robust and generalizable model development. All patients underwent surgical resection of primary lung tumors in this study. Inclusion criteria were as follows: (a) Histopathologically confirmed NSCLC; (b) No prior antitumor therapy before surgery; (c) [^18^F]FDG PET/CT imaging acquired within 8 weeks before surgery; (d) Complete clinical and histopathologic data. Exclusion criteria included: (a) Receipt of neoadjuvant therapy (chemotherapy, radiotherapy, or immunotherapy before PET/CT or surgery); (b) Evidence of distant metastasis on imaging or intraoperative assessment; (c) Incomplete PET/CT imaging or missing clinical/pathologic data; and (d) Poor image quality unsuitable for radiomics analysis, including motion artifacts, low FDG uptake, or segmented tumor volumes < 64 voxels.

### Clinical and pathologic data collection

Demographic and clinical variables included age, sex, smoking status, history of chronic obstructive pulmonary disease, and AJCC TNM stage [26]. PET/CT imaging parameters, including SUVmax, SUVmean, metabolic tumor volume (MTV), and total lesion glycolysis (TLG), were recorded for all patients. Pathological data, including histological subtype, STAS, LVI, and VPI, were confirmed by an experienced pathologist (≥ 10 years of experience). Additionally, the TCIA cohort provided another subset of patients with publicly available imaging and clinical data. Among these, 76 TCIA patients had matched RNA sequencing data. The primary clinical endpoint was progression-free survival (PFS), defined as the time (in months) from surgery to radiologically confirmed recurrence or last follow-up without progression. Recurrence included any local, regional, or distant disease progression based on CT and/or PET/CT. Biopsy of recurrent lesions was not routinely required and was only performed when imaging was equivocal or histologic confirmation was necessary for treatment decisions. All patients had a minimum follow-up duration of one year. Patient characteristics are summarized in Table 1.

**Table 1:** Baselines of patient characteristics and PET parameter distribution

Clinicopathologic characteristics	Final cohort 244 patients n (%)	TCIA set 76 patients n (%)
Age, median (IQR)	64.5 (59, 70)	69.7 (61, 77)
Gender, n (%)		
Female	144 (59%)	63 (82.9%)
Male	100 (41%)	13 (17.1%)
Smoking history, n (%)		
Non‐smoker	68 (27.9%)	12 (15.8%)
Smoker	176 (72.1%)	64 (84.2%)
COPD status, n (%)		NA
Negative	115 (47.1%)	-
Positive	103 (42.2%)	-
T stage, n (%)		
T1+T2	185 (75.8%)	63 (82.9%)
T3+T4	59 (24.2%)	13 (17.1%)
N stage, n (%)		
N0	141 (57.8%)	59 (77.6%)
N1+N2	103 (42.2%)	17 (22.4%)
Stage*, n (%)		
I	92 (37.7%)	32 (42.1%)
II	97 (39.8%)	34 (44.7%)
III	55 (22.5%)	10 (13.2%)
Histology subtype, n (%)		
ADC	178 (73.0%)	55 (75.3%)
SCC	66 (27.0%)	18 (24.7%)
VPI, n (%)		
Negative	174 (71.3%)	53 (72.6%)
Positive	70 (28.7%)	20 (27.4%)
LVI, n (%)		NA
Negative	140 (57.4%)	-
Positive	104 (42.6%)	-
STAS, n (%)		NA
Negative	159 (65.2%)	-
Positive	85 (34.8%)	-
Recurrence, n (%)		
Yes	103 (42.2%)	24 (31.6%)
No	141 (57.8%)	52 (68.4%)
nDmaxC, median (IQR)	0.49 (0.32, 0.69)	0.31 (0.221, 0.50)
nDmaxP, median (IQR)	0.20 (0.15, 0.36)	0.32 (0.18, 0.46)
EPS, median (IQR)	0.34 (0.06, 0.59)	0.52 (−0.22, 0.30)
Tumor SUVmax, median (IQR)	9.5628 (5.84, 17.87)	2.1027 (0.65, 2.48)
Tumor MTV, median (IQR)	16.126 (6.10, 48.05)	15.734 (6.79, 46.55)
Tumor TLG, median (IQR)	68.115 (16.98, 289.4)	8.4152 (2.96, 24.44)
Tumor SUVmean, median (IQR)	3.9735 (2.53, 6.13)	1.5185 (0.34, 0.86)

*ADC* adenocarcinoma, *SCC* squamous cell carcinoma, *COPD* Chronic obstructive pulmonary disease, *MTV* metabolic tumor volume, *TLG* total lesion glycolysis, *VPI* visceral pleural invasion, *LVI* lymphovascular invasion, *STAS* spread through air spaces, *IQR* interquartile range. NA, not applicable. *Based on TNM 8th edition

### Image preprocessing and feature extraction

[^18^F]FDG PET/CT scans were obtained from two centers (Vienna and Budapest) using institutional standard protocols aligned with EANM/EARL guidelines [27]. Tumors were segmented semi-automatically using a 40% SUVmax threshold. Images were resampled and normalized before radiomic feature extraction. A total of 115 PET radiomic features were extracted following IBSI guidelines [28]. Two spatial features, nDmaxC and nDmaxP, were used to derive the EPS, which quantifies the spatial shift of SUVmax within the tumor (Supplementary Fig. 1A). It was defined as:

$$\:Edge\:Proximity\:Score=\frac{nDmaxC-nDmaxP}{nDmaxC+nDmaxP}$$

A higher EPS reflects SUVmax localization toward the tumor edge. Patients were stratified using a geometrically defined and biologically interpretable cutoff (EPS > 0 vs. ≤ 0), where 0 represents equal proximity of SUVmax to the tumor centroid and perimeter. Full preprocessing and feature extraction details are provided in the Supplementary Methods.

Following an 80:20 training–validation split, radiomic features were standardized, and the 20 features were selected using the minimum redundancy maximum relevance (mRMR) algorithm. Eight machine learning models were applied to predict LVI, VPI, and STAS. Feature importance was ranked using SHAP analysis [29, 30]. To assess model robustness, we additionally performed five-fold cross-validation on the full dataset, as detailed in the Supplementary Methods.

### Outcome prediction and exploring biological functions

The prognostic value of EPS was assessed using univariable and multivariable Cox regression based on the final patient cohort. Patients were stratified into high-risk (EPS > 0) and low-risk (EPS ≤ 0) groups using a geometrically defined cutoff. PFS was analyzed using Cox regression and Kaplan–Meier (KM) survival curves. A prognostic nomogram was constructed using independent Cox predictors and validated in the TCIA cohort. Time-dependent area under the curve (AUC) and calibration curves were used to evaluate model performance. Transcriptomic and immune cell infiltration analyses were performed on a subset of TCIA patients with available RNA sequencing data. Full analysis details are provided in the Supplementary Methods.

### Statistical analyses

Continuous variables were summarized as medians with interquartile ranges (IQRs), and categorical variables as counts and percentages. The Mann-Whitney U test was used for comparisons of continuous variables between groups. Spearman correlation was used to assess associations between EPS and selected radiomic features. Machine learning model performance for predicting LVI, VPI, and STAS was evaluated using AUC, sensitivity, and specificity. Feature importance was assessed using SHAP. Prognostic analysis for PFS was conducted using univariable and multivariable Cox regression, with hazard ratios (HRs) and 95% confidence intervals (CIs) reported. Follow-up time was estimated using the reverse KM method. A prognostic nomogram was constructed using independent predictors identified in the final multivariable Cox model and externally validated in the TCIA cohort. Discriminative performance was evaluated using time-dependent AUC at 1-, 3-, and 5-year PFS. All analyses were performed using R (version 4.1.0) and Python (version 3.6). A two-sided *P* value < 0.05 was considered statistically significant.

## Results

### Patient characteristics

Out of 785 screened NSCLC patients, 244 were included in the final cohort (Fig. 1). Baseline characteristics of the final and TCIA cohorts are summarized in Table 1. The median age was 64.5 years; 59% were female; and most had early-stage tumors (T1–T2: 75.8%; N0: 57.8%) and a smoking history (72.1%). Adenocarcinoma was the predominant histology (73%), with frequent presence of VPI (28.7%), LVI (43.6%), and STAS (34.8%). Recurrence occurred in 42.2% of the final cohort (median follow-up: 102 months) and 31.6% in TCIA (median: 41 months). EPS and its components (nDmaxC and nDmaxP) were significantly associated with LVI, VPI, and STAS (*all P* < 0.0001). EPS and nDmaxC were higher, and nDmaxP lower, in NSCLC with invasive features (Supplementary Fig. 1B–D). EPS showed weak correlation with conventional PET features and radiomics (|*r*| < 0.5), supporting its distinct spatial contribution (Supplementary Fig. 2).

### Predictive performance of radiomic models

**Fig. 2:**
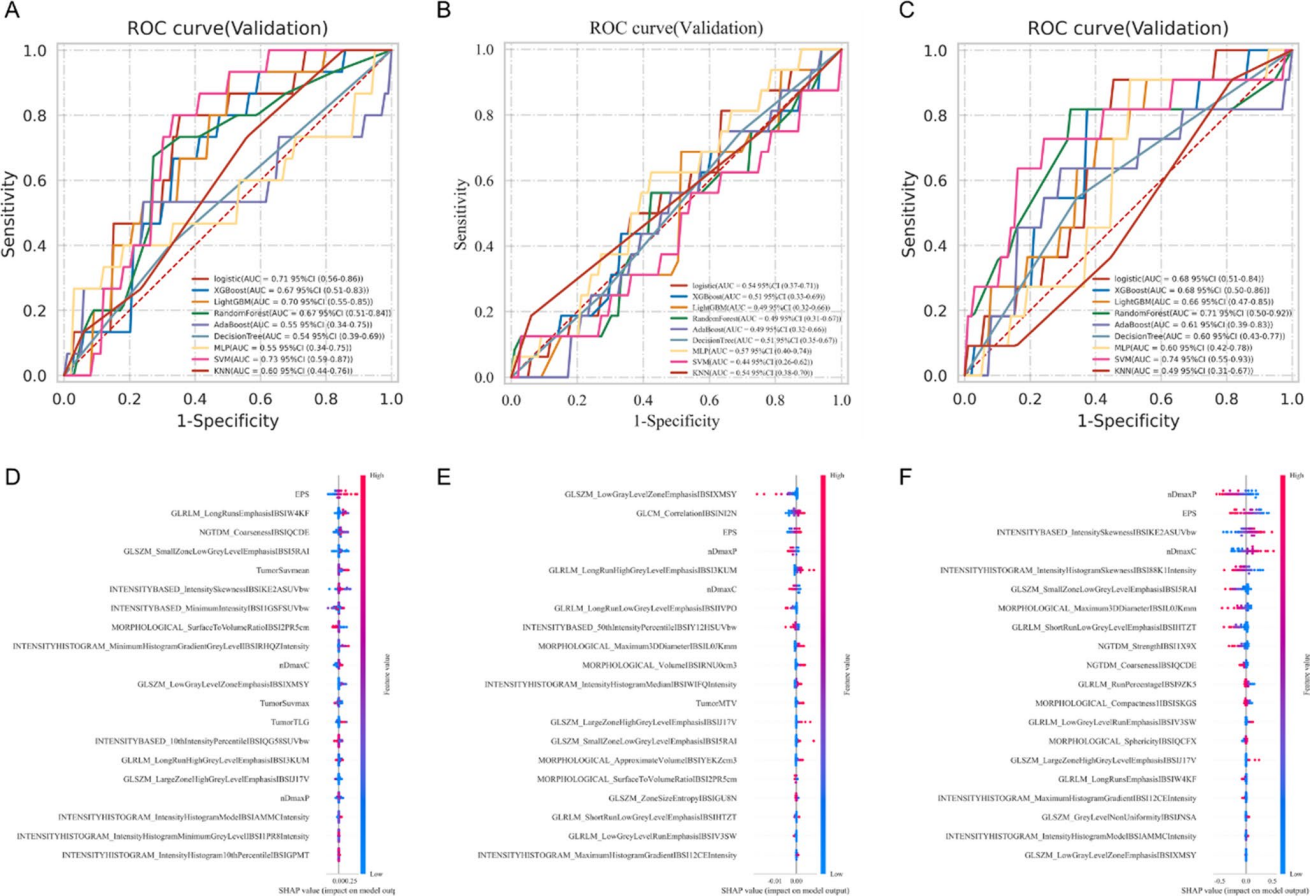
Comparative analysis of machine learning classifiers for predicting histopathologic features in NSCLC. Radiomics-based models were developed to predict the presence of STAS, LVI, VPI using eight supervised machine learning classifiers, including XGBoost, logistic regression, LightGBM, random forest, AdaBoost, decision tree, MLP, and SVM. Panels **A**–**C** present the classification performance of each model in the independent validation cohort, measured by the area under the receiver operating characteristic curve, for STAS (**A**), LVI (**B**), and VPI (**C**). Panels **D**–**F** display the corresponding SHAP summary plots, which illustrate the relative importance of the 20 mRMR-selected radiomic features used for model predictions of STAS (**D**), LVI (**E**), and VPI (**F**), ranked by their mean absolute SHAP values. LVI, lymphovascular invasion; MLP, multilayer perceptron; NSCLC, non–small cell lung cancer; VPI, visceral pleural invasion; STAS, spread through air spaces; SVM, support vector machine; SHAP, Shapley additive explanations

The predictive performance of eight machine learning classifiers for identifying STAS, LVI, and VPI status in the 20% validation cohort is summarized in Fig. 2 and Supplementary Table 1. For STAS prediction (Fig. 2A), the support vector machine (SVM) achieved the highest performance (AUC = 0.73, 95% CI: 0.59–0.87). The extreme gradient boosting (XGBoost) yielded the best discriminative ability for LVI (AUC = 0.61, 95% CI: 0.44–0.77; Fig. 2B), while SVM performed best for VPI classification (AUC = 0.74, 95% CI: 0.55–0.93; Fig. 2C). SHAP analysis identified the EPS among the top five contributors to model predictions across all endpoints (Fig. 2D–F). To further evaluate model performance, we also conducted five-fold cross-validation using the full dataset. The results are presented in Supplementary Fig. 3.

### Prognostic stratification

EPS was evaluated as a predictor of PFS using Cox regression (Supplementary Fig. 4A–B). In univariable analysis, EPS was significantly associated with shorter PFS (HR = 7.650; 95% CI: 3.893–15.036; *P* < 0.001), along with age, TNM stage, MTV, VPI, and other clinical variables (*P* < 0.05). In multivariable analysis, EPS remained an independent predictor (HR = 2.667; 95% CI: 1.205–5.935; *P* = 0.015), along with TNM stage (HR = 2.927; *P* = 0.018), VPI (HR = 1.628; *P* = 0.030), age (HR = 0.975; *P* = 0.030), and tumor MTV (HR = 1.005; *P* = 0.041). nDmaxC (HR = 1.682; *P* = 0.207) and nDmaxP (HR = 0.303; *P* = 0.222) were not significant when tested separately (Supplementary Fig. 4C–D).

**Fig. 3:**
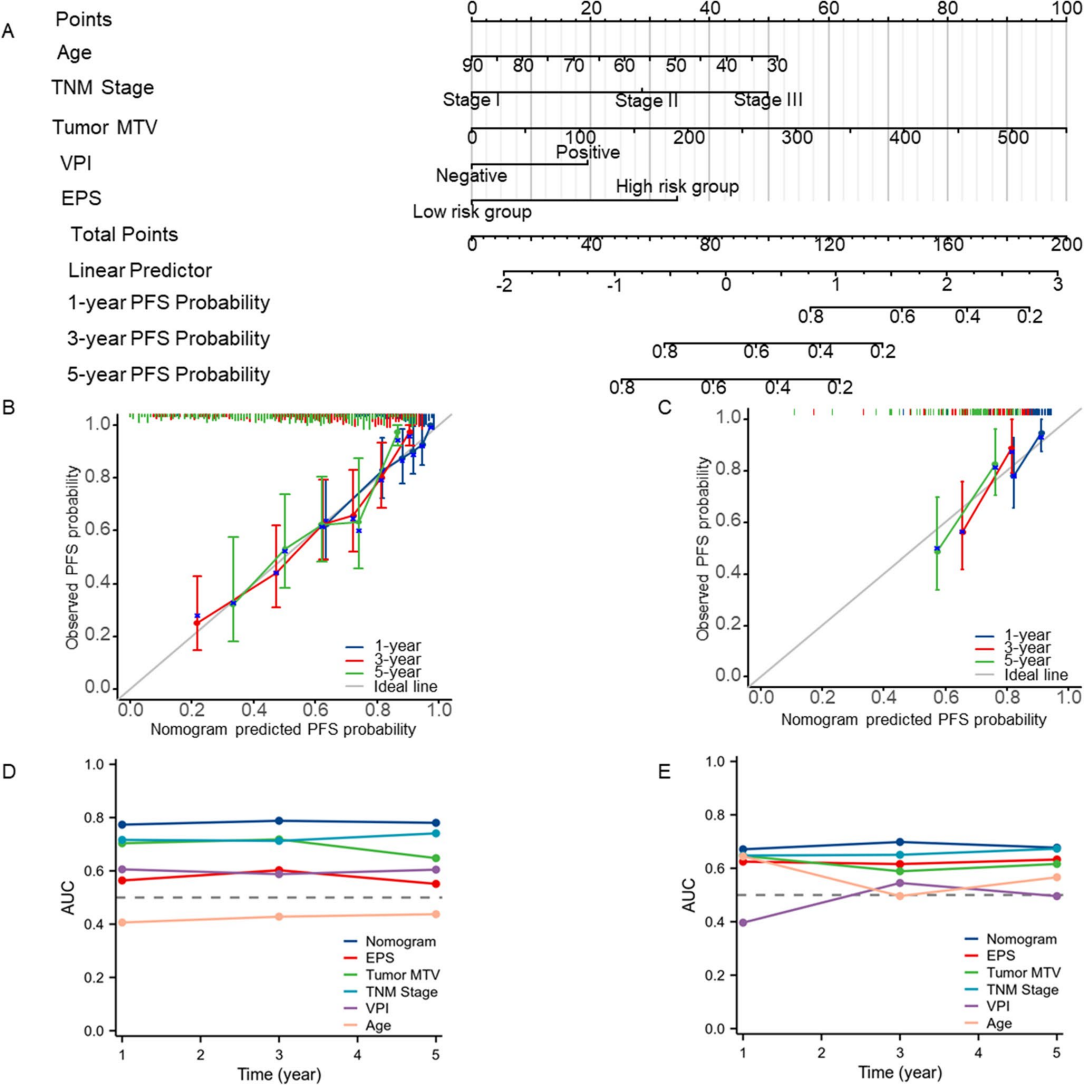
Nomogram for progression-free survival prediction and calibration in the training (Final) and validation (TCIA) Cohorts. (**A**) Nomogram constructed using the training (final) cohort to predict 1-, 3-, and 5-year PFS based on age, TNM stage, MTV, VPI, and the EPS. Each variable contributes to a point total, which maps to predicted PFS probabilities. Calibration plots evaluating nomogram performance in the training (final) cohort (**B**) and the validation (TCIA) cohort (**C**). The x-axis represents nomogram-predicted PFS probabilities; the y-axis shows the actual observed survival derived from Kaplan–Meier estimates. Vertical bars indicate 95% confidence intervals. Time-dependent AUC curves comparing the prognostic performance of the nomogram, EPS, TNM stage, MTV, VPI, and age for predicting 1-, 3-, and 5-year PFS in the training (final) cohort (**D**) and the validation (TCIA) cohort (**E**). PFS, progression-free survival; MTV, metabolic tumor volume; VPI, visceral pleural invasion; EPS, Edge Proximity Score. AUC, area under the curve

**Fig. 4:**
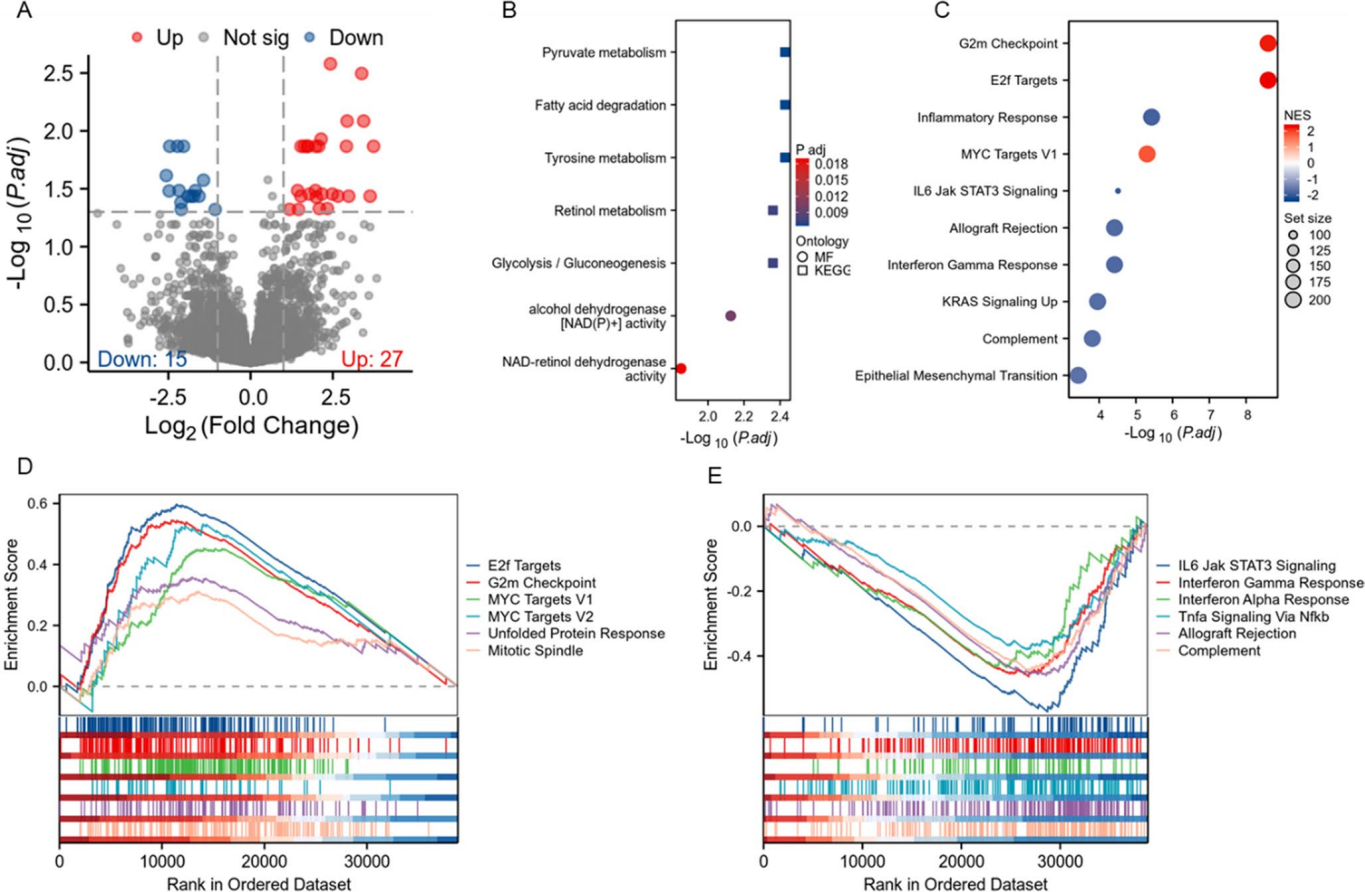
Functional differences between low- and high-risk groups based on Edge Proximity Score using RNA sequencing data (*n* = 76, TCIA set). (**A**) Volcano plot displaying differentially expressed genes between high- and low-risk groups. Significantly upregulated genes in the high-risk group are shown in red (*n* = 27), while downregulated genes in the low-risk group are shown in blue (*n* = 15). (**B**) Bubble plot showing enriched Gene Ontology (GO) and Kyoto Encyclopedia of Genes and Genomes (KEGG) terms among the differentially expressed genes. Dot size indicates the ontology source (MF = molecular function; KEGG = pathway), and color represents adjusted p-values (P.adj, FDR corrected). (**C**) Hallmark gene set enrichment analysis reveals significantly enriched biological pathways in the high-risk group (red, positive normalized enrichment score [NES]) and low-risk group (blue, negative NES). (**D**, **E**) Gene set enrichment plots illustrate hallmark pathways upregulated in the high-risk group (**D**) and in the low-risk group (**E**), with the x-axis representing the rank in the ordered dataset and the y-axis showing the enrichment score

A prognostic nomogram was constructed based on the independent predictors identified in the multivariable Cox model from the training (final) cohort (Fig. 3A). Calibration plots showed good agreement between predicted and observed 1-, 3-, and 5-year PFS in both the training (final) and validation (TCIA) cohorts (Fig. 3B–C). In the TCIA validation cohort, EPS yielded AUCs of 0.63 (95% CI: 0.47–0.78), 0.62 (95% CI: 0.49–0.75), and 0.63 (95% CI: 0.50–0.77) for 1-, 3-, and 5-year PFS, respectively. The nomogram achieved AUCs of 0.67 (95% CI: 0.52–0.83), 0.70 (95% CI: 0.57–0.83), and 0.68 (95% CI: 0.53–0.83) (Fig. 3D–E and Supplementary Table 2). KM analysis showed significantly worse PFS in high-risk (EPS > 0) patients in both cohorts (final: *P* < 0.0001; TCIA: *P* = 0.021; Fig. 4B; Supplementary Fig. 5A). EPS stratified prognosis within stage I and stage II-III subgroups (*P* < 0.0001 and *P* = 0.0099, respectively), supporting its added value beyond TNM staging (Supplementary Fig. 5C–D).

### Biological functions associated with EPS status

To explore the biological underpinnings of the EPS, we analyzed RNA sequencing data from 76 NSCLC patients in the TCIA dataset, comparing high-risk (EPS > 0) and low-risk (EPS ≤ 0) group. We identified 42 differentially expressed genes (27 upregulated, 15 downregulated; adjusted *P* < 0.05; Fig. 4A). Functional enrichment analysis of these DEGs using Gene Ontology (GO) and Kyoto Encyclopedia of Genes and Genomes (KEGG) pathways revealed that genes upregulated in the high-risk group were predominantly involved in pyruvate metabolism, fatty acid degradation, and glycolysis/gluconeogenesis (Fig. 4B). Gene set enrichment analysis (GSEA) using Hallmark gene sets further indicated enrichment of proliferation-related pathways (G2M checkpoint, E2F targets, MYC signaling) in high-risk group, whereas immune-related pathways (interferon α/γ response, IL6-JAK-STAT3, and TNF-α/NF-κB signaling) were downregulated (Fig. 4C–E). Given the immune signature in low-risk group, we profiled immune infiltration using single-sample gene set enrichment analysis (ssGSEA) and microenvironment cell populations (MCP)-counter. The low-risk group showed higher infiltration of neutrophils, immature dendritic cells, NK cells, eosinophils, and mast cells (*P* < 0.05; Supplementary Fig. 6A and Supplementary Table 3). MCP-counter confirmed greater abundance of neutrophils (*P* = 0.030), endothelial cells, monocytic lineages, and myeloid dendritic cells (Supplementary Fig. 6B and Supplementary Table 4).

## Discussion

This study presents and validates the EPS, a novel PET-based spatial imaging feature that quantifies SUVmax displacement toward the tumor edge. In a multi-center cohort of resectable NSCLC, EPS was significantly associated with invasive histopathologic features (STAS, VPI, LVI; all *P* < 0.0001) and ranked among the top predictors in machine learning models. EPS remained an independent prognostic factor for PFS (*P* = 0.015) and stratified outcomes even within TNM stage subgroups. Transcriptomic and immune profiling further revealed that EPS captures biologically distinct tumor phenotypes: tumors with high EPS were enriched in metabolic and proliferative pathways, while low EPS tumors exhibited immune-related activity, including interferon signaling and cytokine response.

Previous studies have highlighted the prognostic relevance of spatial PET features such as the normalized hotspot-to-centroid distance (NHOC) and normalized SUVmax-to-perimeter distance (nSPD) [23, 25]. These metrics have been associated with poor outcomes in lung and breast cancer, and their 3D extensions (NHOC and NHOP) were further validated in advanced NSCLC [26]. EPS builds upon these concepts by integrating both centroid- and perimeter-based distances into a composite metric, enhancing its robustness in capturing spatial metabolic asymmetry. While both nDmaxC and nDmaxP were individually associated with histopathologic invasiveness, neither consistently ranked among the top features across machine learning models for different endpoints, nor were they independently prognostic in multivariable Cox models, emphasizing the additive value of their combination. In multivariable Cox regression, EPS remained an independent predictor of PFS alongside MTV [31], VPI [32], and age [33] all of which are established prognostic markers in NSCLC. In the TCIA validation cohort, the prognostic performance of EPS alone was slightly lower than that of TNM stage, yet EPS still added complementary information. To improve clinical applicability, we constructed a multivariable nomogram incorporating EPS along with TNM stage, MTV, VPI, and age. The nomogram demonstrated superior performance and calibration compared to individual predictors, supporting its utility for individualized PFS risk prediction. Stratification using a geometrically defined cutoff (EPS = 0), representing equal proximity to tumor centroid and edge, further distinguished high-risk patients across cohorts. Although this cutoff is not biologically validated, it is conceptually motivated: EPS = 0 reflects equal proximity of SUVmax to the tumor centroid and edge. This midpoint is consistent with prior spatial imaging biomarkers, such as NHOC and NHOP, which have similarly demonstrated that peripheral SUVmax localization is associated with more aggressive tumor phenotypes.

Although spatial imaging features have previously been associated with prognosis in NSCLC, their biological relevance has remained unexplored. To address this, we performed transcriptomic and immune profiling to explore the EPS. Functional enrichment analyses revealed that tumors with high EPS were enriched in glycolysis, pyruvate metabolism, and fatty acid degradation–pathways commonly associated with metabolic reprogramming and tumor aggressiveness [34–36]. Hallmark gene sets further indicated upregulation of G2M checkpoint, E2F targets, and MYC signaling in high EPS tumors, reflecting enhanced proliferation and cell cycle dysregulation [37, 38]. In contrast, low EPS showed enrichment of immune-related pathways, including interferon signaling, IL6-JAK-STAT3, and TNF-α/NF-κB [39, 40], along with higher infiltration of neutrophils, NK cells, monocytic lineages, and dendritic cells as confirmed by ssGSEA and MCP-counter. These findings suggest that EPS captures biologically distinct tumor states, with low EPS identifying a potentially immune-inflamed phenotype. Notably, neutrophils, known for their dual N1 (anti-tumor) and N2 (pro-tumor) phenotypes, may in this setting reflect a more N1-skewed response, supported by the concurrent enrichment in interferon-driven immunity [41]. These transcriptomic and cellular profiles support its relevance for patient stratification in NSCLC.

This study has limitations. The retrospective design may introduce selection bias and incomplete data. Imaging was acquired across two institutions using different scanners, potentially impacting radiomic reproducibility. However, EPS appeared robust to such variability, particularly compared to high-frequency texture features. Although major motion artifacts were excluded, residual respiratory motion may influence measurements; future phantom-based studies could address this. Transcriptomic analysis was limited to a publicly available subset (*n* = 76), which may reduce statistical power. Finally, although EPS showed consistent prognostic value across internal and external cohorts, larger prospective studies with harmonized imaging protocols are warranted to confirm its clinical utility and generalizability.

## Conclusion

This study presents the EPS, a novel spatial PET-based radiomic feature that captures SUVmax displacement within tumors, as a robust prognostic biomarker in resectable NSCLC. The score demonstrated independent prognostic value for PFS and reflected distinct biological states, with high EPS associated with metabolic and proliferative activity, and low EPS linked to immune-inflamed tumor phenotypes. Transcriptomic and immune cell profiling support the biological interpretability of this imaging feature, highlighting its potential role in non-invasive risk stratification. Future work should focus on validating the EPS in larger prospective cohorts and assessing its value in guiding immunotherapy selection and multimodal treatment strategies.

## Supplementary Information

Below is the link to the electronic supplementary material.

Supplementary Material 1

## Data Availability

The datasets generated or analysed during the current study are available from the corresponding author upon reasonable request.
