# Supplementary material for: Spatial imaging features derived from SUVmax location in resectable NSCLC are associated with tumor aggressiveness
Source: Eur J Nucl Med Mol Imaging. 2025 Aug 21;53(3):1458–68. doi: 10.1007/s00259-025-07528-0 (PMC12860873; doi:10.1007/s00259-025-07528-0)
Supplement: Supplementary file 1 — Supplementary Material 1 [file 259_2025_7528_MOESM1_ESM.docx]

**Spatial imaging features derived from SUVmax location in resectable NSCLC are associated with tumor aggressiveness**

**Supplementary materials**

**Image preprocessing and feature extraction**

[18F]FDG PET/CT scans were obtained from two centers: Vienna General Hospital, using a Siemens Biograph Vision 600 scanner, and the National Korányi Institute of Pulmonology in Budapest, using a Siemens Biograph TruePoint 6 HD system. Patients followed standard clinical preparation protocols, including fasting for ≥6 hours to maintain blood glucose levels below 150 mg/dL. Non-contrast low-dose CT was performed for attenuation correction and anatomical localization of PET findings. PET images were reconstructed using scanner-specific clinical protocols, ensuring consistency with EANM/EARL recommendations [1]. Image intensities were converted to standardized uptake values (SUVs) normalized to body weight. Tumors were segmented semi-automatically using a 40% SUVmax threshold by an experienced nuclear medicine physician (7 years of experience), under the supervision of a senior nuclear medicine specialist (10 years of experience). All images were resampled to a uniform voxel grid (4 × 4 × 4 mm³) and underwent intensity discretization with a fixed bin width of 0.31 SUV (192 gray levels between 0–60 SUV), prior to radiomic feature extraction.

Radiomic features were extracted in LIFEx v7.8.0 according to IBSI guidelines [2]. A total of 115 features were extracted: 4 conventional PET metrics (SUVmax, SUVmean, MTV, TLG), 14 morphological features, 42 first-order intensity features, and 55 texture features (GLCM = 23, GLRLM = 11, NGTDM = 5, GLSZM = 16). Two spatial morphological features–nDmaxC and nDmaxP–were combined to define the EPS, a composite metric designed to capture the distribution of metabolic activity within the tumor (Supplementary Fig. 1A).

**Dataset preparation and machine learning workflow**

A multi-step analysis was conducted to compare the Edge Proximity Score (EPS) with other radiomic features in predicting lymphovascular invasion (LVI), visceral pleural invasion (VPI), and spread through air spaces (STAS) in resectable NSCLC. First, descriptive comparisons were performed between EPS, nDmaxC, and nDmaxP values across present vs. absent cases for each histopathologic feature using Mann–Whitney U tests. Correlations among spatial and radiomic features were visualized using correlograms with absolute Spearman correlation coefficients. The full dataset was randomly split into training (80%) and independent validation (20%) cohorts. Radiomic features were standardized using Z-score normalization, with mean and standard deviation values calculated from the training cohort and applied to both sets. Feature selection was then performed independently within the training cohort using the minimum redundancy maximum relevance (mRMR) algorithm. For each classification task (LVI, VPI, STAS), the top 20 most informative features were selected. Eight supervised machine learning classifiers were trained on the selected features: XGBoost, logistic regression, LightGBM, random forest, AdaBoost, decision tree, multilayer perceptron (MLP), and support vector machine (SVM). Models were optimized and evaluated on the 20% hold-out validation set. To interpret model predictions and assess the influence of individual features, SHAP (Shapley Additive Explanations) values were calculated on the independent validation set. For each model and endpoint, the top five most impactful features were ranked based on mean absolute SHAP value.

**Cross-Validation for Model Robustness**

To evaluate model generalizability and robustness, a separate five-fold cross-validation was conducted on the full dataset. In each fold, the dataset was split into a training (80%) and test (20%) subset. Z-score normalization and mRMR feature selection were performed independently within the training subset. The same eight machine learning models were trained using the selected features and evaluated on the corresponding test fold. This process was repeated across all five folds. Final model performance was summarized using the mean and standard deviation of AUC values across folds. SHAP interpretation was not applied in this cross-validation setting due to variability in selected feature sets across folds.

**Prognostic analysis and Nomogram construction**

Univariable Cox regression was performed for all clinical, imaging, and pathologic variables. Variables with *P* < 0.05 were entered into a multivariable Cox model. A prognostic nomogram was constructed using the final multivariable model derived from the training cohort. The nomogram was validated in the TCIA cohort. Time-dependent AUC values at 1-, 3-, and 5-year PFS were computed to assess discriminative performance. Calibration curves were generated using 200 bootstrap resamples.

**RNA sequencing dataset and preprocessing**

RNA sequencing (RNA-seq) data from 76 patients with non-small cell lung cancer (NSCLC) were obtained from The Cancer Imaging Archive (TCIA) [3], each with paired [¹⁸F]FDG PET/CT imaging and clinical outcome data. Gene expression was downloaded in FPKM format for visualization and in raw count format for differential expression analysis. Patients were stratified into high-risk and low-risk groups based on a geometrically defined cutoff of the Edge Proximity Score (EPS) =0. RNA was extracted from resected tumor tissue.

**Differential gene expression analysis**

Differential expression analysis was conducted using the “DESeq2” package. Genes with an adjusted P value < 0.05 (Benjamini-Hochberg correction) were considered significantly differentially expressed. Volcano plots were generated using the “EnhancedVolcano” package to visualize expression fold-changes and statistical significance between EPS groups.

**Gene set enrichment analysis**

To explore the biological functions of EPS, Gene Set Enrichment Analysis (GSEA) was performed using the “clusterProfiler” package (v4.6.2) with the Kyoto Encyclopedia of Genes and Genomes (KEGG), Gene Ontology (GO) biological processes, and Hallmark gene sets downloaded from the Molecular Signatures Database (MSigDB, v2023.1). The analysis used the pre-ranked gene list based on log₂ fold change, with statistical thresholds of |normalized enrichment score (NES)| > 1 and adjusted P < 0.05 considered significant.

**Immune cell infiltration analysis**

The tumor immune microenvironment was profiled using both ssGSEA (single-sample Gene Set Enrichment Analysis) and MCP-counter algorithms. We performed the

“ssGSEA” package [4] to assess the enrichment scores of immune cells infiltration [5]. The association of immune cells infiltration and image features of EPS and MCP-counter scores was derived using the MCPcounter package in R. EPS-based differences in immune cell infiltration were assessed using the Wilcoxon rank-sum test. All statistical analyses were performed in R (v4.2.2). Boxplots were generated using ggplot2 for visual comparison, and P < 0.05 was considered statistically significant.

**Data visualization**

All transcriptomic and immune analyses were performed in R (v4.2.2). Heatmaps, GSEA plots, and immune cell boxplots were created using ggplot2, pheatmap, and enrichplot.

**References**

1. Boellaard R, Delgado-Bolton R, Oyen WJG, et al. FDG PET/CT: EANM procedure guidelines for tumour imaging: version 2.0. Eur J Nucl Med Mol Imaging 2015; 42: 328–54. https://doi.org/10.1007/s00259-014-2961-x

2. Zwanenburg A, Vallières M, Abdalah MA, et al. The image biomarker standardization initiative: Standardized quantitative radiomics for high-throughput image-based phenotyping. Radiology 2020; 295: 328–38. https://doi.org/10.1148/radiol.2020191145

3. Bakr, S., Gevaert, O., Echegaray, S., et al. (2017). Data for NSCLC Radiogenomics (Version 4) [Data set]. The Cancer Imaging Archive. https://doi.org/10.7937/K9/TCIA.2017.7hs46erv

4. Barbie DA, Tamayo P, Boehm JS, et al. Systematic RNA interference reveals that oncogenic KRAS-driven cancers require TBK1. Nature. 2009;462(7269):108-112. doi:10.1038/nature08460

5. Charoentong P, Finotello F, Angelova M, et al. Pan-cancer Immunogenomic Analyses Reveal Genotype-Immunophenotype Relationships and Predictors of Response to Checkpoint Blockade. Cell Rep. 2017;18(1):248-262. doi:10.1016/j.celrep.2016.12.019

| **Supplementary Fig.1 Definition and predictive relevance of the Edge Proximity Score in NSCLC** |
| --- |
| 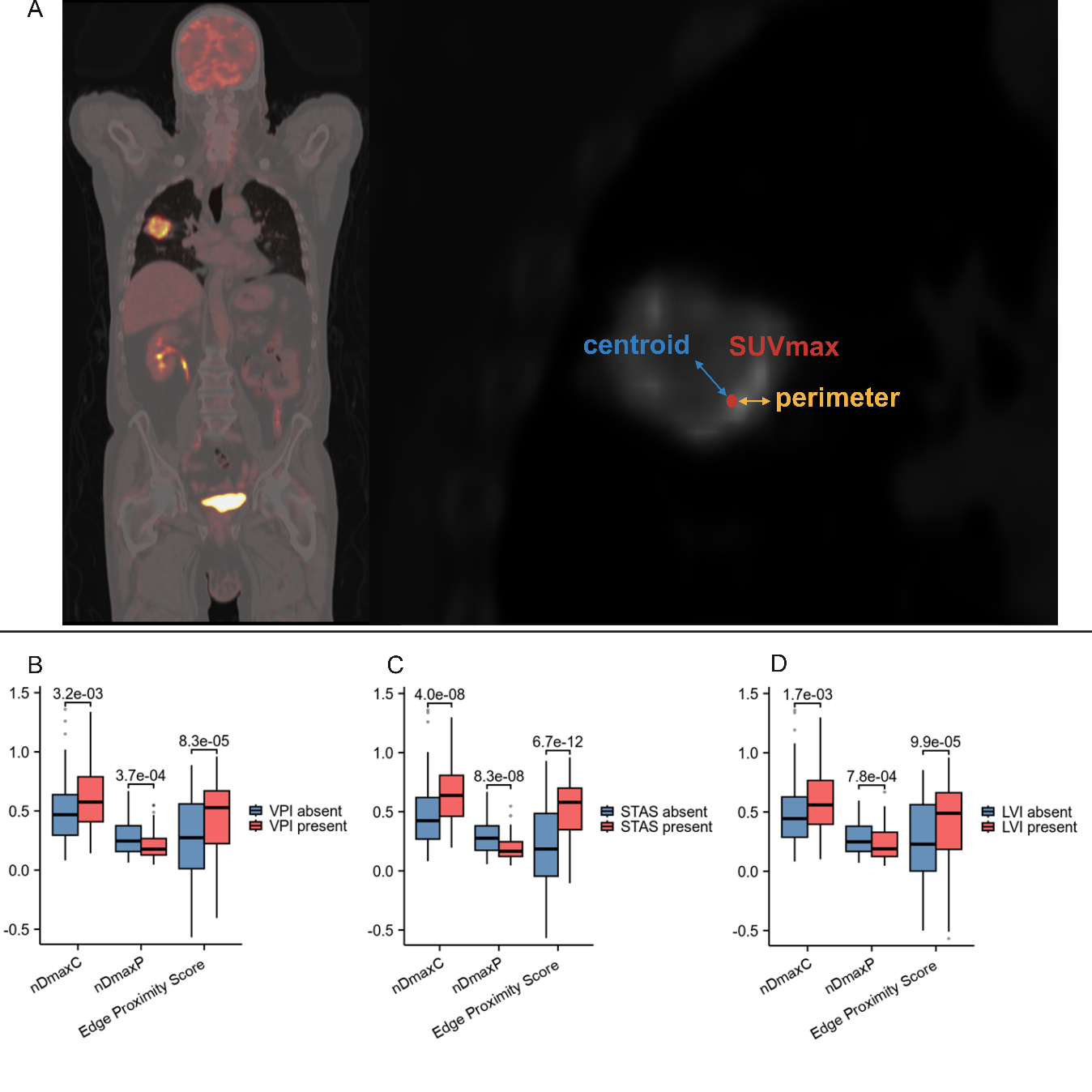 |
| (A) Schematic representation of the EPS derived from [^18^F]FDG-PET/CT. The score quantifies the spatial displacement of metabolic activity (SUVmax) relative to the tumor centroid and perimeter. It is defined as: Edge Proximity Score = (nDmaxC-nDmaxP) / (nDmaxC + nDmaxP), where nDmaxC is the normalized distance from SUVmax to the tumor centroid, and nDmaxP is the normalized distance from SUVmax to the tumor perimeter. (B–D) Boxplots illustrating significantly higher EPS in tumors with aggressive histopathologic features: (B) STAS, (C) VPI, and (D) LVI.  EPS, Edge Proximity Score; nDmaxC, normalized distance from SUVmax to the tumor centroid; nDmaxP, normalized distance from SUVmax to the tumor perimeter; VPI, visceral pleural invasion; LVI, lymphovascular invasion; STAS, spread through air spaces |

| **Supplementary Fig.2 Correlation between Edge Proximity Score and PET-derived radiomic features** |
| --- |
| 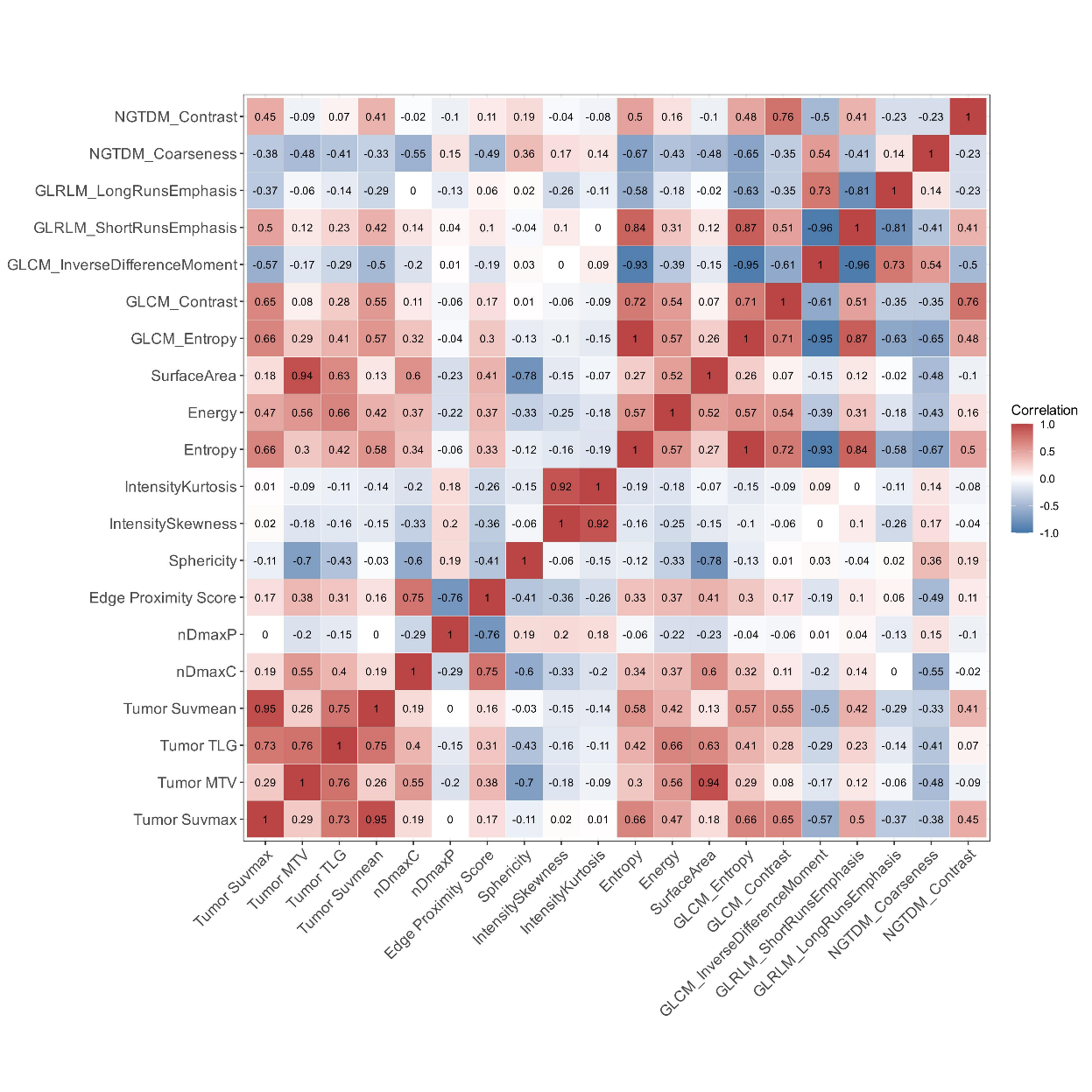 |
| Correlogram illustrating the Spearman correlation coefficients between the EPS and a subset of conventional PET and texture-based radiomic features. Blue tones indicate negative correlations; red tones represent positive correlations. Features are clustered hierarchically based on similarity. nDmaxC, normalized distance from SUVmax to the tumor centroid; nDmaxP, normalized distance from SUVmax to the tumor perimeter; MTV, metabolic tumor volume; TLG, total lesion glycolysis; GLCM, gray-level co-occurrence matrix; GLRLM, gray-level run length matrix; NGTDM, neighborhood gray tone difference matrix. |

| **Supplementary Fig. 3 Five-fold cross-validation AUCs for predicting STAS, LVI, and VPI using radiomics-based models** |
| --- |
| 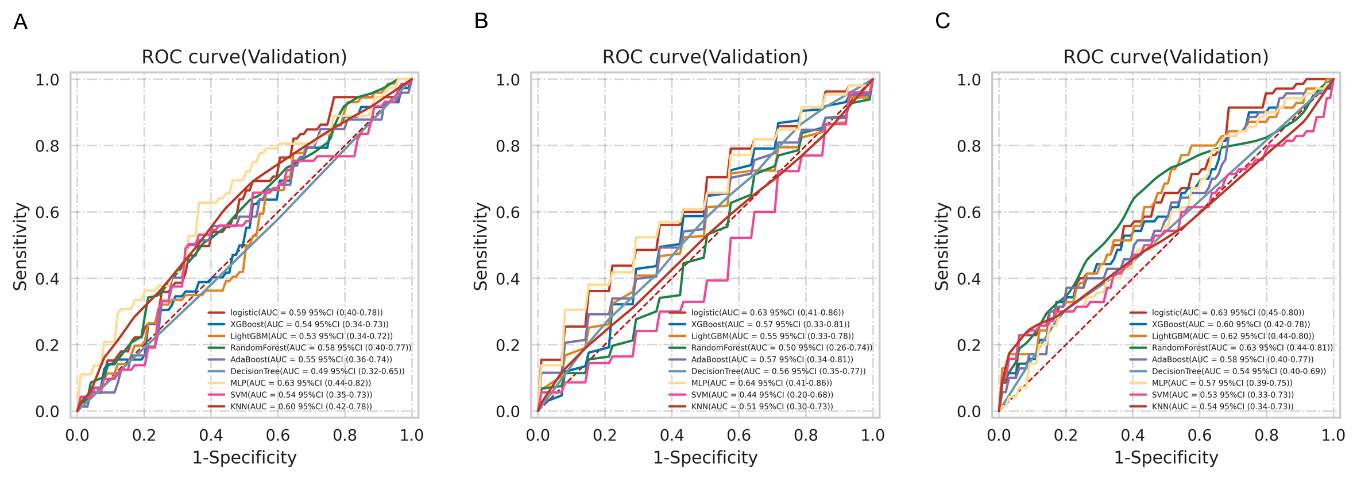 |
| Radiomics-based models were developed to predict the presence of STAS, LVI, and VPI using eight supervised machine learning classifiers: XGBoost, logistic regression, LightGBM, random forest, AdaBoost, decision tree, MLP, and SVM. Panels A–C show the classification performance of each model in the five-fold cross-validation cohort, measured by the area under the receiver operating characteristic curve (AUC), for STAS (A), LVI (B), and VPI (C).  Abbreviations: LVI, lymphovascular invasion; MLP, multilayer perceptron; STAS, spread through air spaces; SVM, support vector machine; VPI, visceral pleural invasion. |

| **Supplementary Fig. 4 Prognostic evaluation of Edge Proximity Score and related spatial features in NSCLC patients** |
| --- |
| 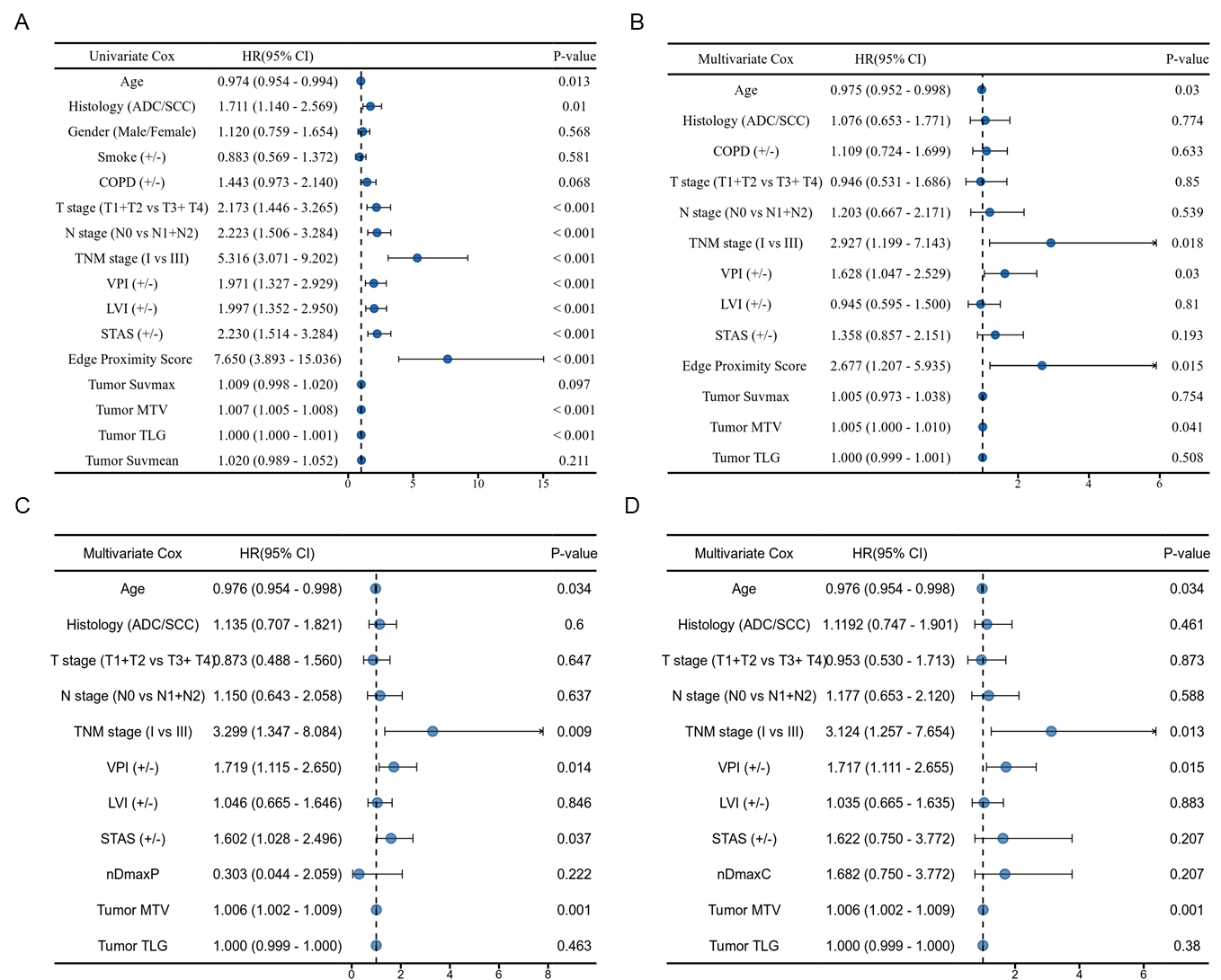 |
| (A) Univariate Cox regression analysis of clinical, pathological, and imaging features for progression-free survival (PFS) in the final cohort. (B) Multivariable Cox regression identifying age, TNM stage, VPI, tumor MTV, and the Edge Proximity Score (EPS) as independent predictors of PFS. (C) Multivariable Cox regression model including nDmaxC (NHOC) instead of EPS. (D) Multivariable Cox regression model including nDmaxP (NHOP) instead of EPS. Hazard ratios (HRs), 95% confidence intervals (CIs), and P-values are shown for each variable. Features were selected based on univariate significance and included in separate multivariable models to evaluate their prognostic contribution. EPS, nDmaxC, and nDmaxP were analyzed independently to assess comparative value.  TNM, tumor–node–metastasis; VPI, visceral pleural invasion; LVI, lymphovascular invasion; STAS, spread through air spaces; MTV, metabolic tumor volume; TLG, total lesion glycolysis; nDmaxC (NHOC), normalized distance from SUVmax to the tumor centroid; nDmaxP (NHOP), normalized distance from SUVmax to the tumor perimeter |

| **Supplementary Fig.5 Kaplan–Meier curves for progression-free survival stratified by Edge Proximity Score and TNM stage.** |
| --- |
| 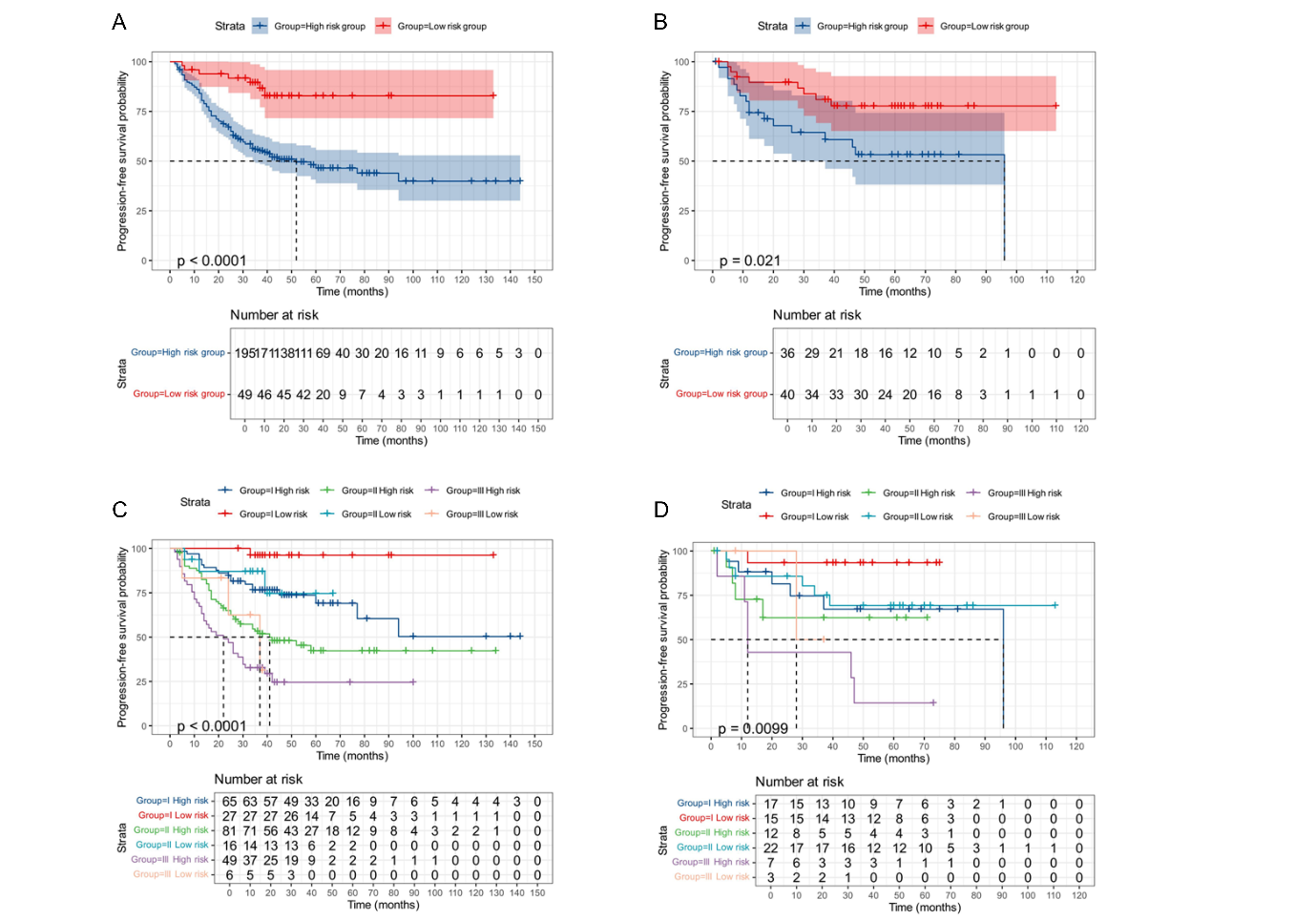 |
| (A) Kaplan–Meier (KM) survival curves comparing PFS between high-risk and low-risk groups (based on Edge Proximity Score > 0 or ≤ 0) in the final cohort. (B) External validation in the TCIA cohort (n = 76) using the same EPS cutoff. (C) Subgroup analysis in the internal cohort, stratified by TNM stage (stage I vs. II–III), demonstrating additive prognostic value of EPS within staging categories. (D) Corresponding TNM-stratified subgroup analysis in the TCIA validation cohort. Shaded areas indicate 95% confidence intervals. Log-rank P values are shown; number at risk and censoring events are indicated below each curve. PFS = progression-free survival; TCIA = The Cancer Imaging Archive; TNM = Tumor node metastasis. |

| **Supplementary Fig.6 Immune cell infiltration differences between high- and low-risk EPS groups** |
| --- |
| 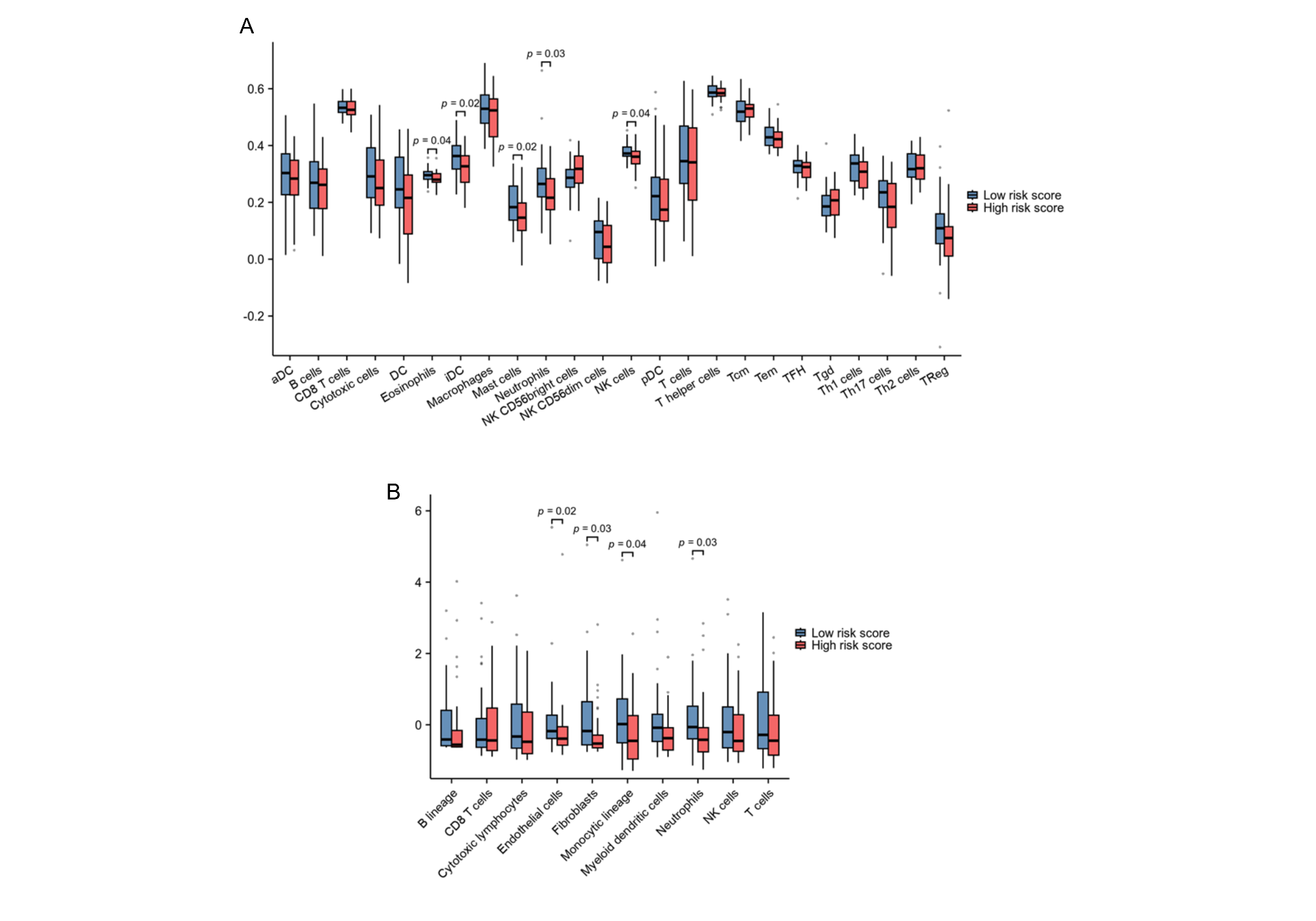 |
| (A) Immune cell abundance estimated by single-sample gene set enrichment analysis (ssGSEA) and (B) Microenvironment cell populations (MCP)-counter |

| **Supplementary Table 1. Predictive performance of machine learning models for STAS prediction in the validation cohort** | | | | | | |
| --- | --- | --- | --- | --- | --- | --- |
| Models | AUC (95%CI) | AC | SN | SP | PPV | NPV |
| LR | 0.71 (0.56-0.86) | 0.69 | 0.47 | 0.79 | 0.5 | 0.77 |
| XGB | 0.67 (0.51-0.83) | 0.69 | 0.13 | 0.94 | 0.5 | 0.71 |
| LGBM | 0.70 (0.55-0.85) | 0.63 | 0.53 | 0.68 | 0.42 | 0.77 |
| RF | 0.67 (0.51-0.84) | 0.65 | 0.47 | 0.74 | 0.44 | 0.76 |
| AB | 0.55 (0.34-0.75) | 0.65 | 0.4 | 0.76 | 0.43 | 0.74 |
| DT | 0.54 (0.39-0.69) | 0.59 | 0.4 | 0.68 | 0.35 | 0.72 |
| MLP | 0.55 (0.34-0.75) | 0.65 | 0.4 | 0.76 | 0.43 | 0.74 |
| **SVM** | 0.73 (0.59-0.87) | 0.65 | 0.8 | 0.59 | 0.46 | 0.87 |
| KNN | 0.60 (0.44-0.76) | 0.53 | 0.73 | 0.44 | 0.37 | 0.79 |
| **Predictive performance of machine learning models for LVI prediction in the validation cohort** | | | | | | |
| Models | AUC (95%CI) | AC | SN | SP | PPV | NPV |
| LR | 0.50 (0.33-0.67) | 0.59 | 0.38 | 0.7 | 0.38 | 0.7 |
| **XGB** | 0.61 (0.44-0.77) | 0.59 | 0.62 | 0.58 | 0.42 | 0.76 |
| LGBM | 0.52 (0.34-0.71) | 0.47 | 0.38 | 0.52 | 0.27 | 0.63 |
| RF | 0.51 (0.34-0.68) | 0.59 | 0.38 | 0.7 | 0.38 | 0.7 |
| AB | 0.51 (0.34-0.68) | 0.53 | 0.31 | 0.64 | 0.29 | 0.66 |
| DT | 0.40 (0.25-0.55) | 0.37 | 0.5 | 0.3 | 0.26 | 0.56 |
| MLP | 0.56 (0.39-0.73) | 0.57 | 0.38 | 0.67 | 0.35 | 0.69 |
| SVM | 0.52 (0.35-0.69) | 0.59 | 0.56 | 0.61 | 0.41 | 0.74 |
| KNN | 0.53 (0.35-0.71) | 0.61 | 0.56 | 0.64 | 0.43 | 0.75 |
| **Predictive performance of machine learning models for VPI prediction in the validation cohort** | | | | | | |
| Model | AUC (95%CI) | AC | SN | SP | PPV | NPV |
| LR | 0.68 (0.51-0.84) | 0.61 | 0.91 | 0.53 | 0.36 | 0.95 |
| XGB | 0.68 (0.50-0.86) | 0.76 | 0.09 | 0.95 | 0.33 | 0.78 |
| LGBM | 0.66 (0.47-0.85) | 0.78 | 0.27 | 0.92 | 0.5 | 0.81 |
| RF | 0.71 (0.50-0.92) | 0.76 | 0.36 | 0.87 | 0.44 | 0.82 |
| AB | 0.61 (0.39-0.83) | 0.67 | 0.55 | 0.71 | 0.35 | 0.84 |
| DT | 0.60 (0.43-0.77) | 0.63 | 0.55 | 0.66 | 0.32 | 0.83 |
| MLP | 0.60 (0.42-0.78) | 0.61 | 0.27 | 0.71 | 0.21 | 0.77 |
| **SVM** | 0.74 (0.55-0.93) | 0.53 | 0.82 | 0.45 | 0.3 | 0.89 |
| KNN | 0.49 (0.31-0.67) | 0.51 | 0.36 | 0.55 | 0.19 | 0.75 |
| VPI, visceral pleural invasion; LVI, lymphovascular invasion; STAS, spread through air spaces; XGB, XGBoost; LR, Logistic Regression; LGBM, Light Gradient Boosting Machine; RF, Random Forest; AB, AdaBoost; DT, Decision Tree; MLP, Multilayer Perceptron; SVM,Support Vector Machine; KNN, K-Nearest Neighbors; AC, Accuracy; SN, sensitivity; SP, specificity, PPV, positive predictive value; NPV,negative predictive value; AUC, area under the curve; NA, not available | | | | | | |

| **Supplementary Table 2. Predictive performance of basic models for 1-year progression-free survival in the training (final) and validation (TCIA) cohorts** | | |
| --- | --- | --- |
|  | Final cohort | TCIA cohort |
| Model | AUC (95CI%) | AUC (95CI%) |
| Nomogram | 0.77(0.69 - 0.86) | 0.67 (0.52 - 0.83) |
| EPS | 0.56(0.51 - 0.62) | 0.63 (0.47 - 0.78) |
| Tumor MTV | 0.70(0.61 - 0.80) | 0.65 (0.46 - 0.84) |
| TNM Stage | 0.72(0.63 - 0.80) | 0.65 (0.49 - 0.81) |
| VPI | 0.61(0.52 - 0.69) | 0.40 (0.29 - 0.51) |
| Age | 0.41(0.31 - 0.51) | 0.64 (0.46 - 0.83) |
| **Predictive performance of basic models for 3-year progression-free survival in the training (final) and validation (TCIA) cohorts** | | |
| Model | Final cohort | TCIA cohort |
| Nomogram | 0.79 (0.73 - 0.85) | 0.70(0.57 – 0.83) |
| EPS | 0.60(0.56 - 0.65) | 0.62(0.49 – 0.75) |
| Tumor MTV | 0.72(0.65 - 0.79) | 0.59(0.43 – 0.75) |
| TNM Stage | 0.71(0.65 - 0.78) | 0.65(0.51 – 0.79) |
| VPI | 0.59(0.52 - 0.65) | 0.55(0.42 – 0.67) |
| Age | 0.43(0.35 - 0.51) | 0.50(0.34 – 0.65) |
| **Predictive performance of basic models for 5-year progression-free survival in the training (final) and validation (TCIA) cohorts** | | |
| Model | Final cohort | TCIA cohort |
| Nomogram | 0.78(0.70 - 0.86) | 0.68(0.53 – 0.83) |
| EPS | 0.55(0.48 - 0.62) | 0.63(0.50 – 0.77) |
| Tumor MTV | 0.65(0.54 - 0.75) | 0.62(0.45 – 0.79) |
| TNM Stage | 0.74(0.65 - 0.83) | 0.67(0.53 – 0.81) |
| VPI | 0.60(0.52 - 0.69) | 0.50(0.37 – 0.62) |
| Age | 0.44(0.33 - 0.55) | 0.57(0.40 – 0.73) |
| MTV, metabolic tumor volume; VPI, visceral pleural invasion; EPS, Edge Proximity Score. AUC, area under the curve | | |

| **Supplementary Table 3. Differences in immune cell abundance between low- and high-risk groups as assessed by ssGSEA** | | | |
| --- | --- | --- | --- |
| Immune Cell Type | Median Difference (High – Low) | 95% CI | P-value |
| aDC | -0.027166 | -0.077793 – 0.023214 | 0.2476 |
| B cells | -0.019096 | -0.070733 – 0.031502 | 0.4223 |
| CD8 T cells | -0.0051654 | -0.019388 – 0.0093684 | 0.5245 |
| Cytotoxic cells | -0.027618 | -0.087563 – 0.02582 | 0.3174 |
| DC | -0.065111 | -0.13077 – 0.0023957 | 0.0587 |
| Eosinophils | -0.011812 | -0.023033 – -0.00041829 | 0.0437 |
| iDC | -0.036864 | -0.065537 – -0.0047713 | 0.0212 |
| Macrophages | -0.02445 | -0.063741 – 0.01164 | 0.1658 |
| Mast cells | -0.049878 | -0.091792 – -0.010445 | 0.0159 |
| Neutrophils | -0.040755 | -0.076788 – -0.0028754 | 0.0337 |
| NK CD56bright cells | 0.03327 | -0.00021099 – 0.063212 | 0.0520 |
| NK CD56dim cells | -0.023797 | -0.067923 – 0.016183 | 0.2190 |
| NK cells | -0.016541 | -0.032224 – -0.00044038 | 0.0384 |
| pDC | -0.02503 | -0.082104 – 0.031698 | 0.4104 |
| T cells | -0.027508 | -0.10019 – 0.03701 | 0.4163 |
| T helper cells | -0.0034553 | -0.017305 – 0.0092206 | 0.4913 |
| Tcm | 0.0063596 | -0.014606 – 0.028293 | 0.5312 |
| Tem | -0.0094016 | -0.03035 – 0.011773 | 0.3703 |
| TFH | -0.011326 | -0.030712 – 0.0050143 | 0.1595 |
| Tgd | 0.0067329 | -0.018233 – 0.035745 | 0.5728 |
| Th1 cells | -0.024938 | -0.04955 – 0.00057872 | 0.0520 |
| Th17 cells | -0.034451 | -0.078224 – 0.0081073 | 0.1130 |
| Th2 cells | -0.0036494 | -0.029073 – 0.024168 | 0.8160 |
| TReg | -0.032954 | -0.076301 – 0.010999 | 0.1307 |

| **Supplementary Table 4. Differences in immune cell abundance between low- and high-risk groups as assessed by the MCP-counter** | | | |
| --- | --- | --- | --- |
| Immune Cell Type | Median Difference (High – Low) | 95% CI | P-value |
| B lineage | -0.080979 | -0.21875 – 0.0019415 | 0.0573 |
| CD8 T cells | -0.063146 | -0.2715 – 0.17556 | 0.5728 |
| Cytotoxic lymphocytes | -0.17774 | -0.45695 – 0.076333 | 0.1361 |
| Endothelial cells | -0.2269 | -0.45208 – -0.04269 | 0.0224 |
| Fibroblasts | -0.2166 | -0.49035 – -0.015311 | 0.0328 |
| Monocytic lineage | -0.39296 | -0.75399 – -0.021959 | 0.0365 |
| Myeloid dendritic cells | -0.25096 | -0.47995 – 0.0089496 | 0.0601 |
| Neutrophils | -0.346 | -0.65874 – -0.023158 | 0.0295 |
| NK cells | -0.22942 | -0.56973 – 0.06292 | 0.1130 |
| T cells | -0.23986 | -0.60863 – 0.10195 | 0.1504 |
